# Factors influencing patients’ choice of clinic at Inanda, KwaZulu-Natal

**DOI:** 10.4102/phcfm.v13i1.2968

**Published:** 2021-09-22

**Authors:** Zethembiso C. Hlongwa, Saajida Mahomed

**Affiliations:** 1 KwaZulu-Natal Department of Health, University of KwaZulu-Natal, Durban, South Africa; 2 Department of Laboratory Medicine, College of Health Science, University of KwaZulu-Natal, Durban, South Africa

**Keywords:** patients’ choice, structural indicator and process indicators, clinic overcrowding, Inanda C CHC

## Abstract

**Background:**

In South Africa, patients are meant to attend the clinic close to their place of residence. However, patients often choose which clinic to attend, which results in overcrowding.

**Aim:**

This study aimed to investigate the structural and process factors influencing patients’ choice to attend a community health centre (CHC) in KwaZulu-Natal, South Africa.

**Setting:**

The study was conducted at the Inanda C Community Health Centre (CHC).

**Methods:**

Systematic random sampling was used to select study participants. A structured questionnaire was used to collect socio-demographic data and assess the factors influencing patients’ choice to attend this CHC.

**Results:**

There were 400 patients who participated. The commonest structural indicator that patients agreed on as the reason they attend Inanda C CHC was because it has enough medication (126, 73.3%). There was a significant difference in the proportion of patients who agreed that seeing a doctor instead of nurse was a reason for attending this clinic with 118 (68.6%) patients from within the catchment area and 170 (74.6%) from outside the catchment area. The commonest process indicators that patients from within and outside the catchment area agreed on as reasons for attending Inanda C CHC were ‘the doctor or nurse explains my sickness and treatment to me’ and ‘I get good quality of care’.

**Conclusion:**

The structural and process indicators that influence patients’ choice of clinic may need to be improved at other clinics in this area in order to decrease the overcrowding at this clinic.

## Introduction

The responsibility of government is to ensure access to affordable healthcare to all citizens. Access to healthcare comes with the responsibility on the healthcare provider to meet the needs of the patients. The choice of healthcare provider is influenced by both patient factors and healthcare provider characteristics. Healthcare provider characteristics are quality indicators that may be categorised as structural, process or outcome.^1^ Structural indicators refer to the organisation of healthcare and the capacity and infrastructure to deliver quality healthcare for patients.^1^ Structural indicators include the availability and accessibility of healthcare providers, type and size of the healthcare facility, experience and qualification of staff and cost of treatment.^2^ Process indicators measure the extent to which healthcare providers give clients specific services that are consistent with recommended guidelines for care.^3^ The process indicators include staffs’ communication skills, staffs’ attitude towards patients, availability of information at the healthcare facility, continuity of care, patients’ waiting time and quality of treatment received.^2^ Outcome indicators measure the effects of care provided by the healthcare facility and may be captured by patients’ satisfaction with healthcare received or mortality rates at the healthcare facility.^1^ Reports have shown that outcome indicators are not usually taken into consideration when a patient selects a healthcare facility to attend.^2^ This is likely because indicators such as mortality rate at a specific healthcare facility are not readily available to patients. Structural and process indicators have been found to be more important in influencing patients’ choices of which healthcare facility to attend.^2,3^

In South Africa, a major challenge within the health sector is the inequality in the distribution of infrastructure, financial and human resources between private and public healthcare facilities. The private health sector serves less than 20% of the population and the public health sector serves the majority of South Africans.^4^ Healthcare facilities in the public sector are insufficiently staffed and equipped.^5,6^ This skew of the distribution of healthcare resources has a negative impact on access to healthcare in the public sector. The resources that are allocated to healthcare facilities in the public sector are meant to cater to the needs of the community in the catchment area for each healthcare facility. When patients attend healthcare facilities that are outside their catchment area, it results in overcrowding, increased waiting times, shortage of medication and surgical supplies and overburdened staff in the affected healthcare facility.

Studies have shown that whilst distance to the clinic is an important determinant in clinical usage, there is an interplay between demographic, social and other quality factors.^7^ In a study conducted in Karen Park Clinic, Pretoria North it was found that although 80% of the 334 respondents visited their nearest clinic at least once, 54.6% reported that they would not return to that clinic.^8^ The reasons cited for not wanting to return to their nearest clinic were mainly healthcare provider’s structural and process indicators and included long queues and waiting time, rude staff and lack of medication.^8^

In South Africa, in the province of KwaZulu-Natal there are 11 health districts. The allocation of resources to healthcare facilities is managed at the district level. In Ethekwini Health District, there are issues with overcrowding at Inanda C Community Health Centre (CHC) with reported underused CHC approximately 10 km away.^9^ It is important to understand the healthcare provider factors that influence patients’ choice of clinic to attend, as these factors can be addressed by the healthcare facility and district management teams.

The aim of this study was to determine the proportion of patients who attend Inanda C CHC from outside the catchment area and investigate the factors influencing patients’ choice to attend this clinic.

## Methods

### Study design

This was an analytic, cross-sectional study.

### Setting

The study was conducted at Inanda C CHC, which serves a catchment population of approximately 95 000 people living in the areas of Newtown C, Ezimangweni, Bhambayi, Nhlungwane and Mzomusha. Services at the clinic include maternal and child health, mental health service, human immunodeficiency virus (HIV) and acquired immunodeficiency syndrome (AIDS), tuberculosis and general outpatient services and emergency care, pharmacy and X-ray services.

### Study population

The study population were adult patients (≥ 18 years) attending the General Outpatient Department at Inanda C CHC during the month of February 2019.

### Study sample

Systematic random sampling was used. The first study participant was selected randomly from the queue and thereafter every third person was selected. Patients who were referred to the clinic from another healthcare facility were excluded. Based on a conservative estimate that 30% of patients attending this clinic were from outside the catchment area, a sample size of 384 was estimated to achieve a statistical power of 80% to detect a small-medium effect size of 0.16 with significance level (alpha) of 0.05.

### Data collection

A structured questionnaire was used to collect socio-demographic data (age, gender, employment status, area of residence) and assess healthcare provider factors influencing patients’ choice to attend this clinic. Additional data collected related to time taken to travel to the clinic, mode of transport and the reason for attending the clinic. A subset of questions on patients’ reasons for not attending a clinic closer to their home was administered to those patients who reported to reside outside the Inanda C CHC catchment area. The questionnaire was developed in English and translated to IsiZulu and then translated back to English to ensure that meaning of the questions remained the same. The questionnaire was piloted amongst 50 participants to ensure that the questions were sufficient to meet the study objectives. Statements relating to structural and process indicators were provided and a four-point Likert scale was used to assess the participant’s response. The participant had to select between the options ‘strongly agree’, ‘agree’, ‘disagree’ or ‘strongly disagree’ for each of the statements. The questionnaire was administered by the investigator who marked the participants’ responses on the questionnaire.

### Data analysis

The responses ‘agree’ and ‘strongly agree’ were combined and categorised as agree and the same process was followed for the responses ‘disagree’ and ‘strongly disagree’. The area of residence of a patient was classified as either from the Inanda C CHC catchment area or outside the catchment area. Continuous variables were expressed as a mean and compared using the student’s *t*-test. Categorical variables were compared using Pearson’s chi-square test and Fisher’s exact test. A binary logistic regression model was used to determine if there is an association between the patient’s profile and the quality indicators and also with the choice of healthcare facility. Multivariate analyses were conducted to assess whether age, gender, employment status and living in the catchment area were associated with the structural and process indicators. All statistical analyses were performed using Statistical Package for the Social Sciences (SPSS version 20.0; IBM Corporation, Armonk, United States). Results were considered significant for *p* < 0.05.

### Ethical consideration

The ethical approval for this study was received from University of KwaZulu-Natal Biomedical Research Ethics Committee (BE680/18). Gatekeeper permission was obtained from the KwaZulu-Natal Provincial Health Research and Ethics Committee (KZ_201812_020). All participants signed an informed consent form before participating in the study.

## Results

### Socio-demographic characteristics

A total of 400 participants were included in the study. The majority of participants were female (242, 60.5%). The mean age for males was 47.8 years (standard deviation [s.d.] = 19.1) and the mean age for females was 44.7 years (s.d. = 18.0), *p* = 0.1. More than a third of the sample were unemployed (*n* = 142, 35.5%) or pensioners (*n* = 136, 34 %) (Table 1).

**TABLE 1:** Socio-demographic profile of patients and reasons for attending Inanda C Community Health Centre, 2019.

Variable	Lives within catchment area		Lives outside of catchment area		*p*
	*n*	%	*n*	%	
**Age category**					
18–39	82	47.7	100	43.9	0.492
40–59	35	20.3	42	18.4	-
60+	55	32.0	86	37.7	-
**Gender**					
Male	69	40.1	89	39.0	0.45
Female	103	59.9	139	61.0	-
**Employment status**					
Employed	23	13.4	26	11.4	0.218
Unemployed	57	33.1	85	37.3	-
Self-employed	6	3.5	2	0.9	-
Pensioner	54	31.4	82	36.0	-
Student	32	18.6	33	14.5	-
**Receiving social grant**					
Yes	111	64.5	157	68.9	0.211
No	61	35.5	71	31.1	-
**Reason for attending clinic**					
Illness	151	89.9	184	79.7	< 0.004
Accident	17	10.1	46	20.3	-

### Proportion of patients from outside the clinic catchment area

More than half the sample (*n* = 228, 57.0%) reported to reside outside the clinic catchment area. A significantly larger proportion of patients who lived outside the clinic catchment area reported to be attending the clinic because of an accident (*n* = 46, 20.3%) compared with patients who lived within the clinic catchment area (*n* = 17, 10.1%) (Table 1). As expected, patients from within the clinic catchment area were significantly more likely to have walked to the clinic compared with patients from outside the clinic catchment area. In addition, the travel time to the clinic for patients who lived outside the clinic catchment area was significantly longer compared with patients who lived within the clinic catchment area.

#### Reasons patients from outside the Inanda C community health centre catchment area choose not to attend clinics closer to their area of residence

The lack of doctors, insufficient number of doctors and long waiting time to see a doctor were the commonest reasons (*n* = 226, 99.1%) that patients cited for not attending a clinic closer to their place of residence (Table 2). Patients also reported process indicators such as the lack of medication and long queues (*n* = 225, 98.7%) at their nearest clinics.

**TABLE 2:** Reasons patients from outside the Inanda C Community Health Centre catchment area choose not to attend clinics closer to their area of residence (*n* = 228).

Variable	Agree	
	*n*	%
**Structural indicators**		
*I choose not to attend my nearest clinic because:*		
There are not enough nurses	222	97.4
There are not enough doctors	226	99.1
It takes too long to see a doctor	226	99.1
I do not get to see a doctor	226	99.1
Nurses are not good at their job	216	94.7
Doctors are not good at their job	209	91.7
**Process indicators**		
*I choose not to attend my nearest clinic because:*		
The staff is unfriendly	215	94.3
They do not have enough medication	225	98.7
They have long queues	225	98.7
Nurses or doctor do not explain my sickness or treatment to me	212	93.0

### Structural and process indicators influencing patients’ choice to attend Inanda C community healthcare

The commonest structural indicator that patients agreed on as the reason they attend Inanda C CHC was because it has enough medication (126, 73.3%) (Table 3). Amongst patients who lived within the clinic catchment area, this was followed by ‘there are enough doctors’, ‘the doctors are good at their jobs’ and ‘the nurses are good at their jobs’ (122, 70.9%). Amongst patients who lived outside the clinic catchment area, the second and third most common structural indicators that patients agreed on as the reason for attending the clinic were ‘the doctors are good at their jobs’ (174, 76.3%), and ‘there are enough doctors’ (173, 75.9%). There was a significant difference in the proportion of patients who agreed that consulting to see a doctor instead of nurse was a reason for attending Inanda C CHC with 118 (68.6%) patients from within the catchment area and 170 (74.6%) from outside the catchment area agreeing with this statement.

**TABLE 3:** Comparison of structural indicators influencing patients’ choice to attend Inanda C Community Health Centre, 2019.

I choose to attend Inanda C Community Health Centre because:	Lives within catchment area		Lives outside catchment areas		*p*
	*n*	%	*n*	%	
**There are enough nurses**					**0.458**
Agree	119	69.2	160	70.2	-
Disagree	53	30.8	68	29.3	-
**There are enough doctors**					**0.159**
Agree	122	70.9	173	75.9	-
Disagree	50	29.1	55	24.1	-
**It does not take long to see a doctor**					**0.115**
Agree	118	68.6	170	74.6	-
Disagree	54	31.4	58	25.4	-
**I get to see a doctor instead of nurse**					**0.009**
Agree	93	54.1	151	66.2	-
Disagree	79	44.9	77	33.8	-
**The nurses here are good at their job**					**0.213**
Agree	122	70.9	171	75.0	-
Disagree	50	29.1	57	25.0	-
**The doctors here are good at their job**					**0.136**
Agree	122	70.9	174	76.3	-
Disagree	50	29.1	54	23.7	-
**They have enough medication**					**0.135**
Agree	126	73.3	179	78.5	-
Disagree	46	26.7	49	21.5	-

The most common process indicators that patients from within the catchment area and patients from outside the catchment area agreed on as reasons for attending Inanda C CHC was ‘the doctor or nurse explains my sickness and treatment to me’ (163, 94.8% and 221, 96.9%, respectively) followed by ‘I get good quality of care’ (160, 93.0% and 218, 95.6%, respectively) (Table 4). There were significantly higher proportions of patients who lived outside the clinic catchment area who agreed with the indicators on short queues, consulting a doctor instead of a nurse and short waiting time.

**TABLE 4:** Comparison of process indicators influencing patients’ choice to attend Inanda C Community Health Centre, 2019.

I choose to attend Inanda C Community Health Centre because:	Lives within catchment area		Lives outside catchment areas		*p*
	*n*	%	*n*	%	
**I get good quality of care**					**0.183**
Agree	160	93.0	218	95.6	-
Disagree	12	7.0	10	4.4	-
**They have short queues**					**0.019**
Agree	110	64.0	169	74.1	-
Disagree	62	36.0	59	25.9	-
**The staff have a good attitude**					**0.054**
Agree	150	87.2	211	92.5	-
Disagree	22	12.8	17	7.5	-
**The waiting time to see the doctor or nurse is not long**					**0.026**
Agree	148	86.0	211	92.5	-
Disagree	24	14.0	17	7.5	-
**The doctor or nurse explains my sickness and treatment to me**					**0.201**
Agree	163	94.8	221	96.9	-
Disagree	9	5.2	7	3.1	-
**The staff are friendly**					**0.035**
Agree	113	65.7	170	74.6	-
Disagree	59	34.3	58	25.4	-

There was no association between gender and the structural or process indicators. Younger patients (18–39 years) were less likely to agree with the structural indicators and with the process indicator on the doctor or nurse explaining the condition to the patient. There was no association between employment status and the indicators linked to waiting time and short queues (results not shown).

## Discussion

This is the first reported study that assessed patients’ reasons for attending a CHC in the public health sector in KwaZulu-Natal. The high proportion of females (60.5%) is in contrast to the general population of Inanda township where 52.0% are female. Whilst it has been shown that females tend to seek healthcare more frequently than males, it must also be observed that more than half of the females attending Inanda C CHC were from outside the clinic catchment area.^10,11^ The high proportion of unemployed patients is in keeping with the poor socio-economic conditions in Inanda and surrounding townships.^12^ This finding is also aligned to the profile of patients who attend public healthcare facilities where most patients are unemployed and do not have private medical insurance.^13^

More than half of the patients in this study lived outside the clinic catchment area. This is in contrast to findings from a study carried out in Pretoria where 80% of participants reported to attend healthcare facilities closest to them.^8^ The high proportion of patients from outside the clinic catchment area may be because of the ease of access to Inanda C CHC, which is located along a main road. Other clinics in the Inanda township are not along main roads and are primary healthcare clinics, which offer less services than a CHC.

The structural indicators that were found to be contributory factors for patients to attend Inanda C CHC (availability of enough medication, doctors and nurses being good at their job and enough medical and nursing staff) may also be because this clinic being a CHC as opposed to a primary healthcare clinic. In South Africa, primary healthcare clinics are staffed with clinical nurse practitioners and medical doctors who visit the clinic once a week, whereas at CHCs, medical doctors are available every day, with a wider range of services and medication available. Moreover, the rehabilitation team consists of a physiotherapist, occupational therapist and speech therapist who visit the CHC at least once a week. In addition, the CHC offers a 24-h emergency service and has X-ray facilities, both of which are not offered at a primary healthcare clinics.^14^ Previous studies in South Africa have shown that the primary healthcare clinics are insufficiently staffed.^14,16^ Similar observations have been reported in Europe and Tanzania where availability of staff, services provided and reliable access to medication were significant contributors to patients’ choice of healthcare facility.^5,6^ In the province of Tehran in the Islamic Republic of Iran, it was reported that structural indicators contribute highly in attracting patients to a healthcare facility.^17^

The most common process indicators that contributed to patients choosing to attend Inanda C CHC were related to the staff (attitude and explaining the condition to the patient) and quality of care including short waiting times. In Europe similar observations were made where explaining medical condition and medication to the patient had a positive influence on their choice of health facility.^5^ This empowerment of patients with knowledge about their condition may result in improved prognosis for the patient as they are likely to be more adherent to their treatment regimen.^15^ Short waiting time has been shown to be a significant predictor in patients’ choice of healthcare facility in Pretoria, as well as in Nigeria.^3,8^ Similar findings have been reported in Europe, the Netherlands and United Kingdom in 2015 where patients who were more concerned about waiting time were less likely to attend healthcare facilities near their place of residence.^5^ Good quality of care and good staff attitude were also found to be positive predictors of choice of healthcare facility in Nigeria.^18^ Furthermore, the study in Tehran also found that staff attitude and poor quality of care had a negative effect on the patient’s choice of a health facility.^17^ In Nigeria patients indicated that they preferred a clinic that was not overcrowded and had a good staff-to-patient ratio.^3^ In addition a cross-sectional study that was carried out in Nigeria in 2013, it was concluded that the choice of healthcare provider is determined by the quality of service provided by the healthcare facility, the attitude of staff and their skills.^3^ In Tanzania, it was found that women preferred reliable access to drugs and equipment and respectful staff.^6^

When comparing the structural and process indicators amongst patients from within and outside Inanda C CHC catchment area, patients from outside the catchment area were more likely to cite short queues, waiting time, staff friendliness and attitude as reasons for attending this clinic. This is in contrast to studies in the United States and United Kingdom that have shown that patients are influenced by financial reasons.^19^ As this study focused on clinics in the public sector that provides free services, financial factors are less likely to be contributory factor. It appears that patients are more influenced by quality of care and being able to see a doctor instead of a nurse. These structural and process indicators were also cited as reasons patients did not attend their nearest clinics, suggesting that the clinics in their areas of residence are lacking in these indicators. Similar findings were reported in Pretoria where patients chose to attend the Karen Park Clinic as they were happy with the staff attitude and reported lack of medication and bad staff attitude as reasons for not attending their nearest clinic.^8^

Interestingly, we did not find an association between employment and selecting short queues and waiting time as reasons for attending the clinic. It is possible that these patients had taken a day off work and were therefore not in a hurry to return to work. We also found no association between gender and any of the structural or process indicators. This in contrast to findings from Nigeria and Tanzania where women preferred skilled professional and reliable access to drugs and equipment and respectful staff who are attentive over other healthcare facility features.^3,6^

One of the limitations of this study is that it was a quantitative study, so we were unable to explore further on patients’ reasons for choosing to attend Inanda C CHC. Factors such as social stigma, particularly for diseases such as HIV have been reported as influencing patients to attend clinics away from home.^20^ However, as the study was conducted at the general outpatient department, social stigma is less likely to be a contributory factor. In addition, we used a questionnaire with preconceived responses that may have been leading the participants response to the question. A comparative qualitative study comparing reasons why patients choose to attend different clinics would be more informative. As the questionnaire was administered by the principal investigator, there may have been an element of social desirability bias with patients reporting positively on how they perceived the care at the clinic. The results of this study have limited generalisability as it was conducted in a single healthcare facility in an urban setting.

## Conclusion

A large proportion of patients attending Inanda C CHC are not from the clinic catchment area. Staff attitude and quality of care are important factors that patients consider when selecting which clinic to attend. Further research at clinics in the surrounding areas is warranted to assess the structural and process indicators that may need to be improved in order to decrease the overcrowding at this clinic.

## References

[1] Victoor A, Delnoij DMJ, Friele RD, Rademakers JJ. Determinants of patient choice of healthcare providers: A scoping review. BMC Health Serv Res. 2012;12(1):272. 10.1186/1472-6963-12-272 22913549 PMC3502383

[2] Tenkorang EY. Health provider characteristics and choice of health care facility among Ghanaian health seekers. Heal Syst Reform. 2016;2(2):160–170. 10.1080/23288604.2016.1171282 31514639

[3] Uchendu OC, Ilesanmi OS, Olumide AE. Factors influencing the choice of health care providing facility among workers in a local government secretariat in south western Nigeria. Ann Ibadan Postgrad Med [serial online]. 2013[cited 2020 Nov 28];11(2):87–95. Available from: https://www.ncbi.nlm.nih.gov/pmc/articles/PMC4111062/# PMC4111062 25161426

[4] Release S. General household survey 2016 [homepage on the Internet]. 2018 [cited 2020 Nov 28]. Available from: http://www.statssa.gov.za/

[5] Laverty AA, Dixon A, Millett C. Do patients’ information requirements for choice in health care vary with their socio-demographic characteristics? Health Expectations. 2015;18(5):1127–1138. 10.1111/hex.12086 23711113 PMC5060873

[6] Kruk ME. Women’s preferences for place of delivery in rural Tanzania: A population-based discrete choice experiment. Am J Public Heal. 2009;99(9):1666–1673. 10.2105/AJPH.2008.146209 PMC2724466 19608959

[7] Frank T, Victoria H, Justus B, Geoffrey S. New approaches to spatially analyse primary health care usage patterns in rural South Africa, Trop Med Int Health. 2001;6(10):826–838. 10.1046/j.1365-3156.2001.00794.x 11679131

[8] Masango-Makgobela AT, Govender I, Ndimande JV. Reasons patients leave their nearest healthcare service to attend Karen Park Clinic, Pretoria North. Afr J Prim Health Care Fam Med. 2013;5(1):559. 10.4102/phcfm.v5i1.559

[9] Hattingh T. District Health Plans 2015/2016 – eThekwini Health District [homepage on the Internet]. 2015 [cited 2020 Nov 28]. Available from: http://www.kznhealth.gov.za/Strategic/DHP/2015-16/eThekwini.pdf

[10] Song J, Chang RW, Manheim LM, Dunlop DD. Gender differences across race/ethnicity in use of health care among medicare-aged Americans. J Wom Health. 2006;15(10):1205–1213. 10.1089/jwh.2006.15.1205 17199461

[11] Hunt K, Adamson J, Hewitt C, Nazareth I. Do women consult more than men? A review of gender and consultation for back pain and headache. J Health Serv Res Policy. 2011;16(2):108–117. 10.1258/jhsrp.2010.009131 20819913 PMC3104816

[12] James D, McGill A, Muller G, Muller K, Skinner D. eThekwini Municipality economic review 2006/2007. eThekwini Municipality Economic Development Department. Durban: eThekwini Municipality Economic Development Department; 2006.

[13] Young M. Private vs. public healthcare in South Africa [homepage on the Internet]. Honors theses. 2016 [cited 2020 Nov 28]. p. 2741. Available from: https://scholarworks.wmich.edu/honors_theses/2741

[14] Dookie S, Singh S. Primary health services at district level in South Africa: A critique of the primary health care approach. BMC Fam Pract. 2012;13:67. 10.1186/1471-2296-13-67 22748078 PMC3403923

[15] Becker MH, Maiman LA. Strategies for enhancing patient compliance. J Community Health. 1980;6:113–135. 10.1007/BF01318980 7204635

[16] Tshililo AR, Mangena-Netshikweta L, Nemathaga LH, Maluleke M. Challenges of primary healthcare nurses regarding the integration of HIV and AIDS services into primary healthcare in Vhembe district of Limpopo province, South Africa. Curationis. 2020;42(1):6. 10.4102/curationis.v42i1.1849 PMC6417489 30843402

[17] Bahadori M, Teymourzadeh E, Ravangard R, Nasiri A, Raadabadi M, Alimohammadzadeh K. Factors contributing towards patient’s choice of a hospital clinic from the patients’ and managers’ perspective. Electron Physician. 2016;8(5):2378–2387. 10.19082/2378 27382448 PMC4930258

[18] Chizoma NM, Tobiloba OO, Ndikwonta CA. Factors influencing the choice of health care provider during childbirth by women in Ibadan, Oyo State, Nigeria. Int J Caring Sci [serial online]. 2017[cited 2020 Nov 28];10(1):1–511. Available from: www.internationaljournalofcaringsciences.org

[19] Wijewickrama AKA. Simulation analysis for reducing queues in mixed-patients’ outpatient department. Int J Simul Model. 2006;5(2):56–68. 10.2507/IJSIMM05(2)2.055

[20] Wolf HT, Halpern-Felsher BL, Bukusi EA, et al. ‘It is all about the fear of being discriminated [against] … the person suffering from HIV will not be accepted’: A qualitative study exploring the reasons for loss to follow-up among HIV-positive youth in Kisumu, Kenya. BMC Public Health. 2014;14:1154. 10.1186/1471-2458-14-1154 25377362 PMC4232620

